# Supplementary material for: Comparative and integrated analysis of plasma extracellular vesicle isolation methods in healthy volunteers and patients following myocardial infarction
Source: J Extracell Biol. 2022 Nov 23;1(11):e66. doi: 10.1002/jex2.66 (PMC11080728; doi:10.1002/jex2.66)
Supplement: Supplementary file 1 — Supporting Information [file JEX2-1-e66-s001.pptx]

## Slide 1
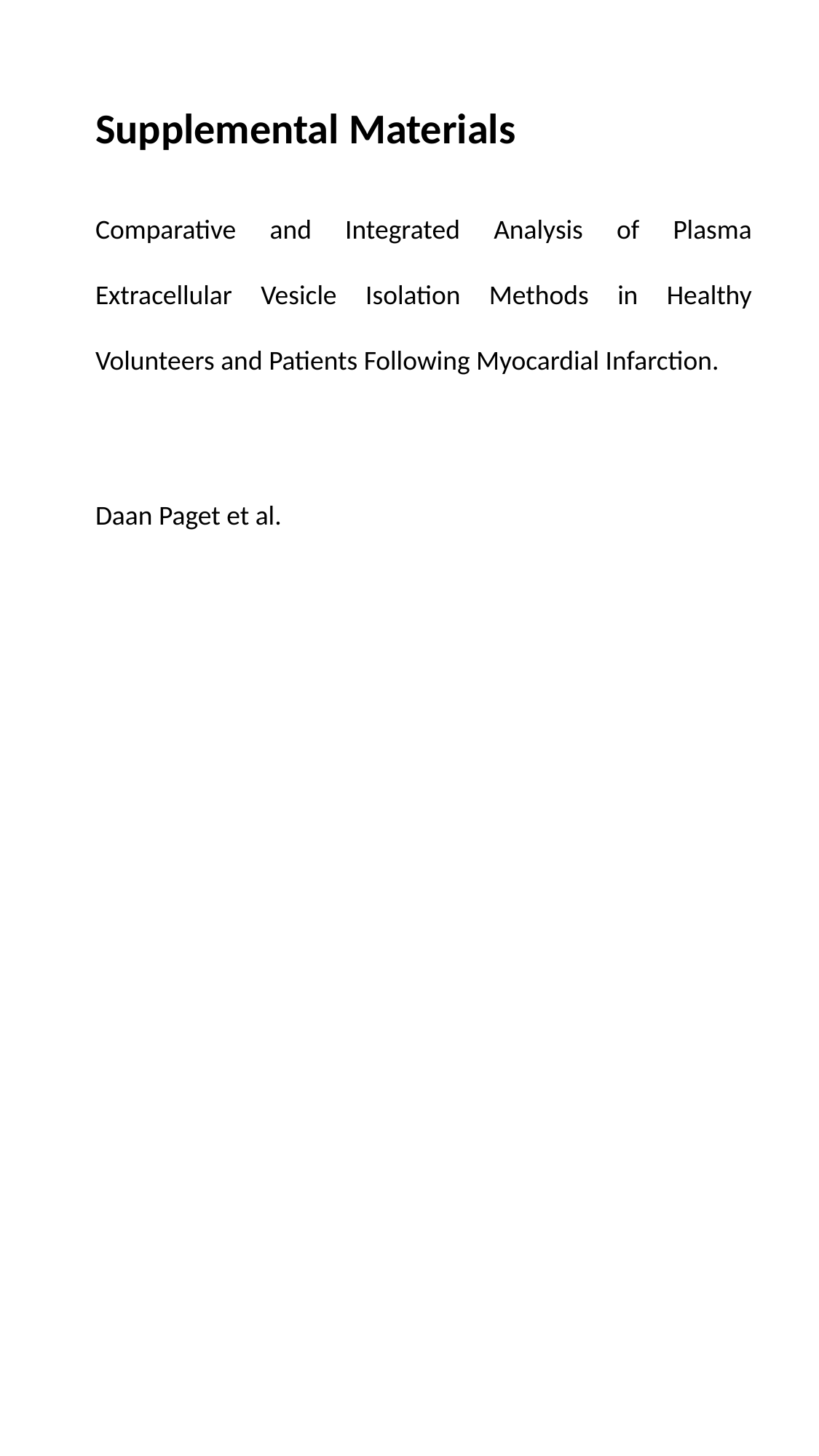

Supplemental Materials
Comparative and Integrated Analysis of Plasma Extracellular Vesicle Isolation Methods in Healthy Volunteers and Patients Following Myocardial Infarction.
Daan Paget et al.

## Slide 2
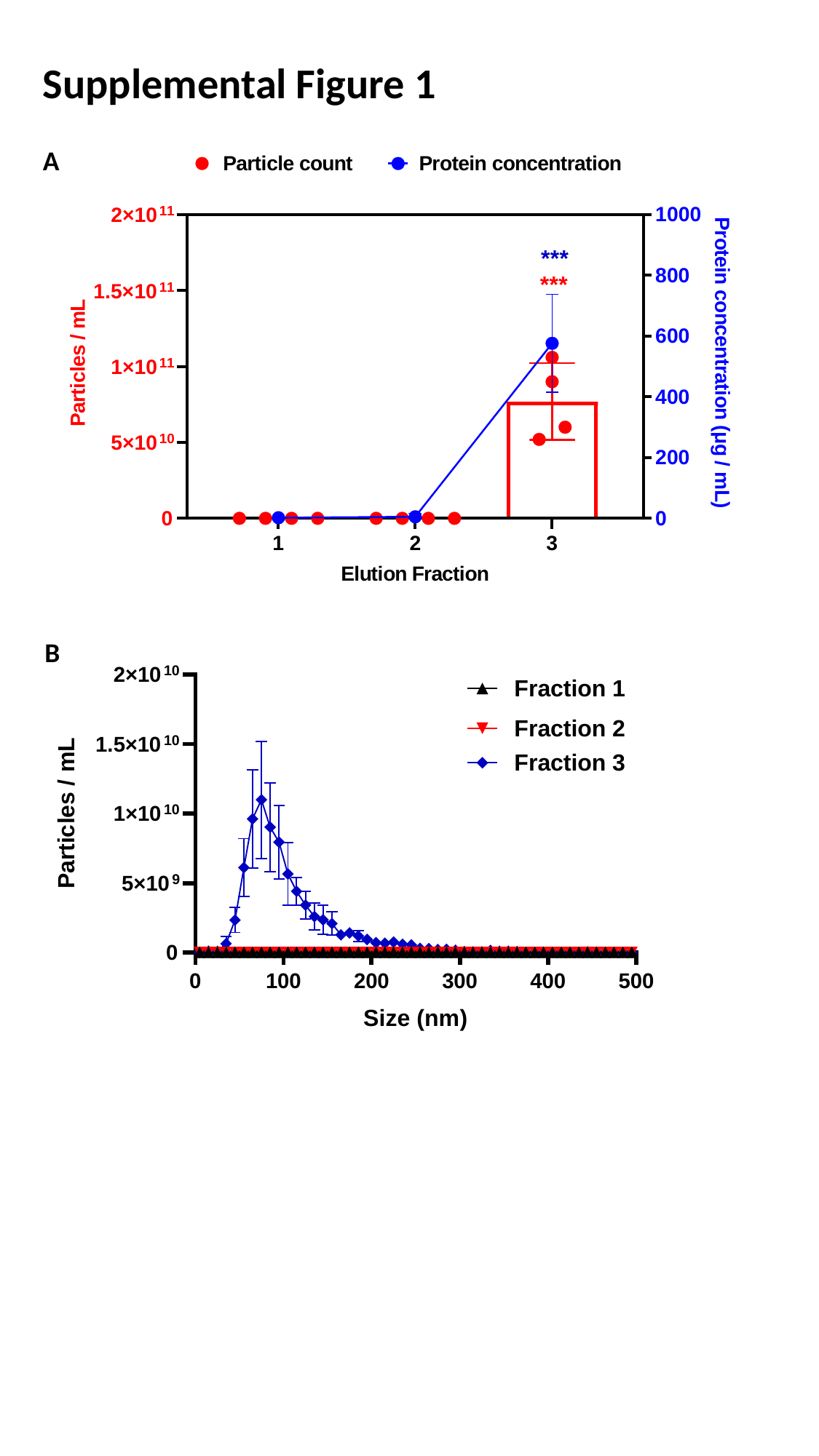

Supplemental Figure 1
A
B

## Slide 3
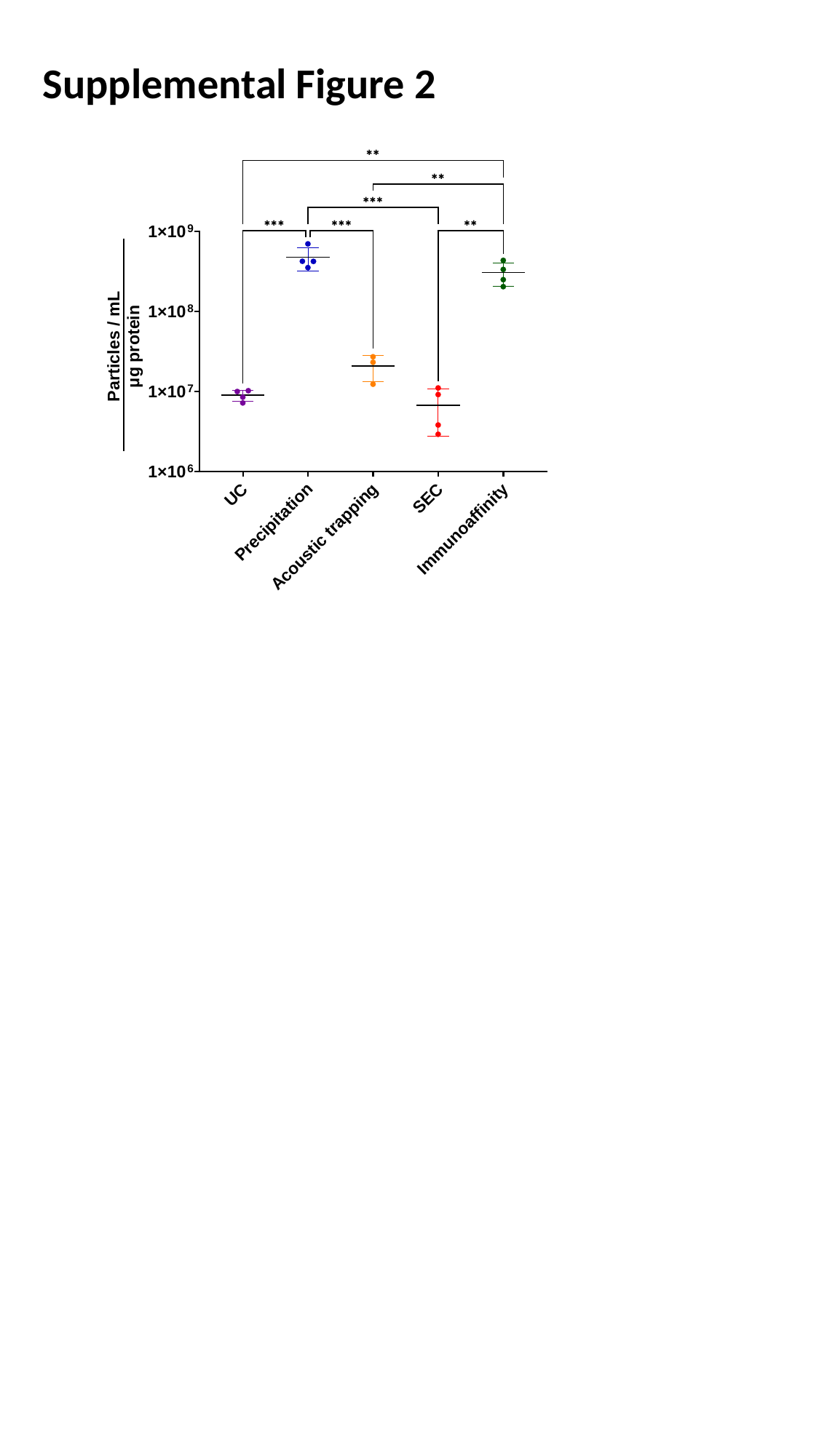

Supplemental Figure 2

## Slide 4
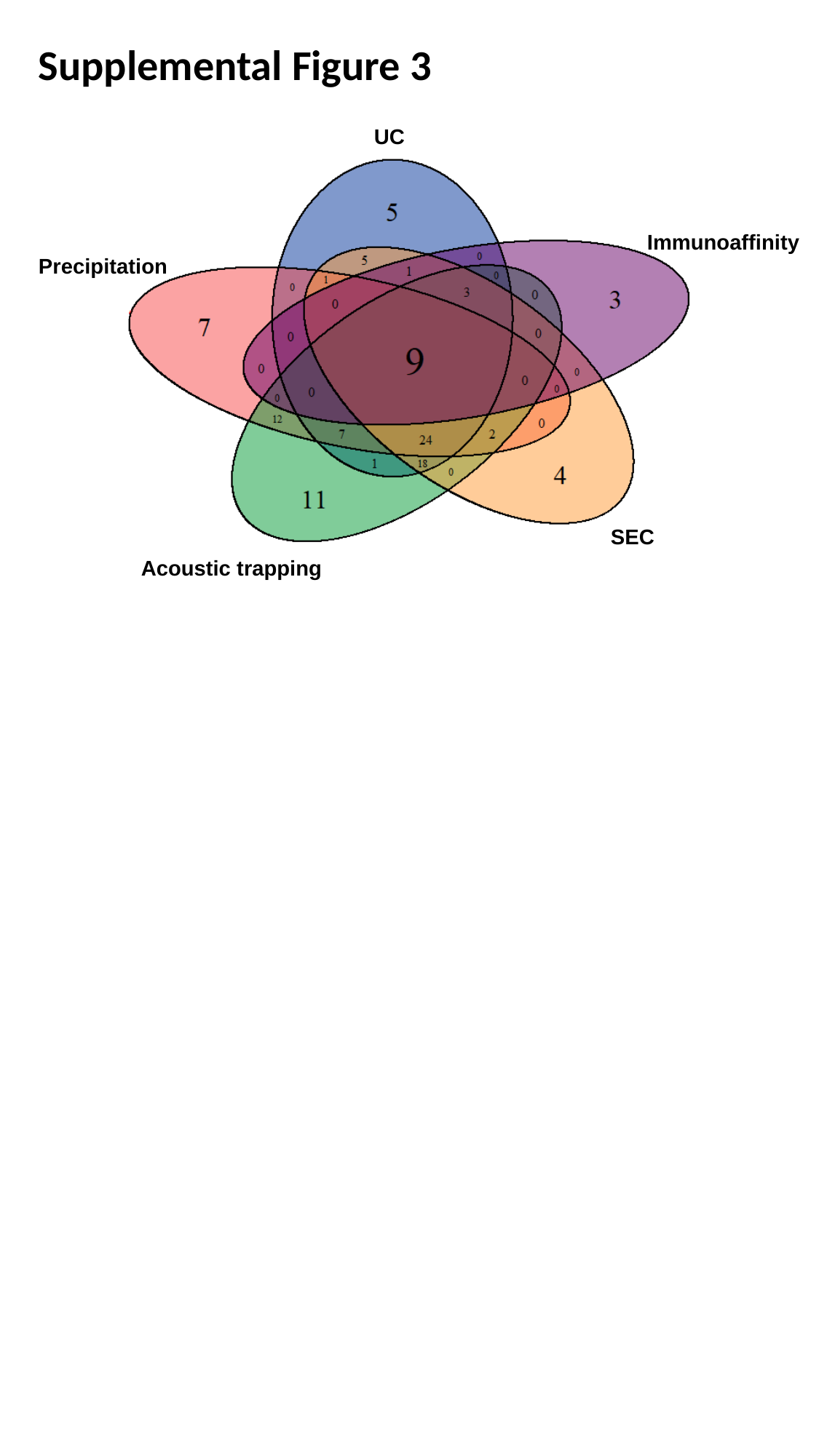

Supplemental Figure 3
UC
Immunoaffinity
Precipitation
SEC
Acoustic trapping

## Slide 5
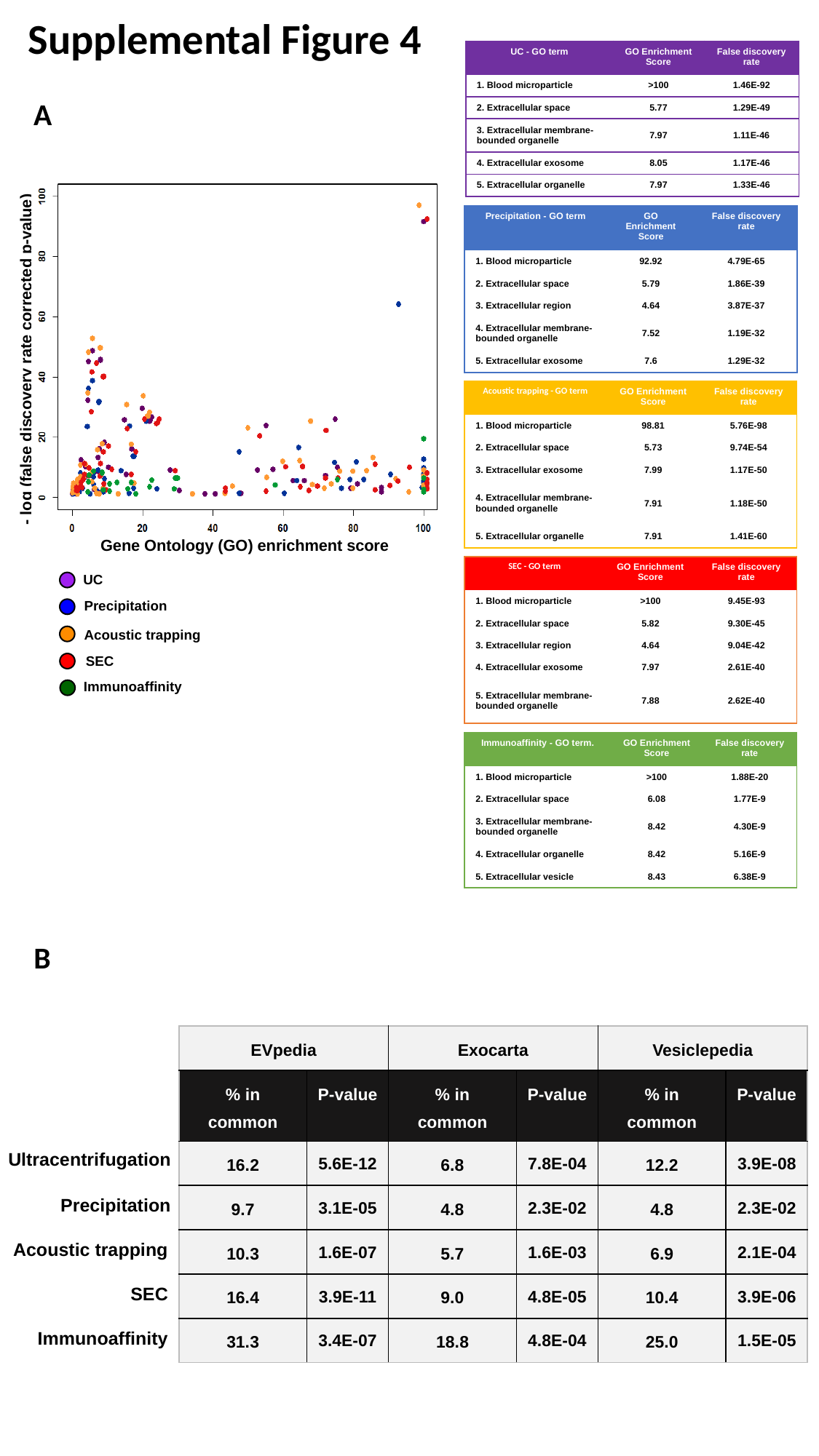

Supplemental Figure 4
| UC - GO term | GO Enrichment Score | False discovery rate |
| --- | --- | --- |
| 1. Blood microparticle | >100 | 1.46E-92 |
| 2. Extracellular space | 5.77 | 1.29E-49 |
| 3. Extracellular membrane-bounded organelle | 7.97 | 1.11E-46 |
| 4. Extracellular exosome | 8.05 | 1.17E-46 |
| 5. Extracellular organelle | 7.97 | 1.33E-46 |
A
| Precipitation - GO term | GO Enrichment Score | False discovery rate |
| --- | --- | --- |
| 1. Blood microparticle | 92.92 | 4.79E-65 |
| 2. Extracellular space | 5.79 | 1.86E-39 |
| 3. Extracellular region | 4.64 | 3.87E-37 |
| 4. Extracellular membrane-bounded organelle | 7.52 | 1.19E-32 |
| 5. Extracellular exosome | 7.6 | 1.29E-32 |
- log (false discovery rate corrected p-value)
| Acoustic trapping - GO term | GO Enrichment Score | False discovery rate |
| --- | --- | --- |
| 1. Blood microparticle | 98.81 | 5.76E-98 |
| 2. Extracellular space | 5.73 | 9.74E-54 |
| 3. Extracellular exosome | 7.99 | 1.17E-50 |
| 4. Extracellular membrane-bounded organelle | 7.91 | 1.18E-50 |
| 5. Extracellular organelle | 7.91 | 1.41E-60 |
Gene Ontology (GO) enrichment score
| SEC - GO term | GO Enrichment Score | False discovery rate |
| --- | --- | --- |
| 1. Blood microparticle | >100 | 9.45E-93 |
| 2. Extracellular space | 5.82 | 9.30E-45 |
| 3. Extracellular region | 4.64 | 9.04E-42 |
| 4. Extracellular exosome | 7.97 | 2.61E-40 |
| 5. Extracellular membrane-bounded organelle | 7.88 | 2.62E-40 |
UC
Precipitation
Acoustic trapping
SEC
Immunoaffinity
| Immunoaffinity - GO term. | GO Enrichment Score | False discovery rate |
| --- | --- | --- |
| 1. Blood microparticle | >100 | 1.88E-20 |
| 2. Extracellular space | 6.08 | 1.77E-9 |
| 3. Extracellular membrane-bounded organelle | 8.42 | 4.30E-9 |
| 4. Extracellular organelle | 8.42 | 5.16E-9 |
| 5. Extracellular vesicle | 8.43 | 6.38E-9 |
B
| EVpedia | | Exocarta | | Vesiclepedia | |
| --- | --- | --- | --- | --- | --- |
| % in common | P-value | % in common | P-value | % in common | P-value |
| 16.2 | 5.6E-12 | 6.8 | 7.8E-04 | 12.2 | 3.9E-08 |
| 9.7 | 3.1E-05 | 4.8 | 2.3E-02 | 4.8 | 2.3E-02 |
| 10.3 | 1.6E-07 | 5.7 | 1.6E-03 | 6.9 | 2.1E-04 |
| 16.4 | 3.9E-11 | 9.0 | 4.8E-05 | 10.4 | 3.9E-06 |
| 31.3 | 3.4E-07 | 18.8 | 4.8E-04 | 25.0 | 1.5E-05 |
Ultracentrifugation
Precipitation
Acoustic trapping
SEC
Immunoaffinity

## Slide 6
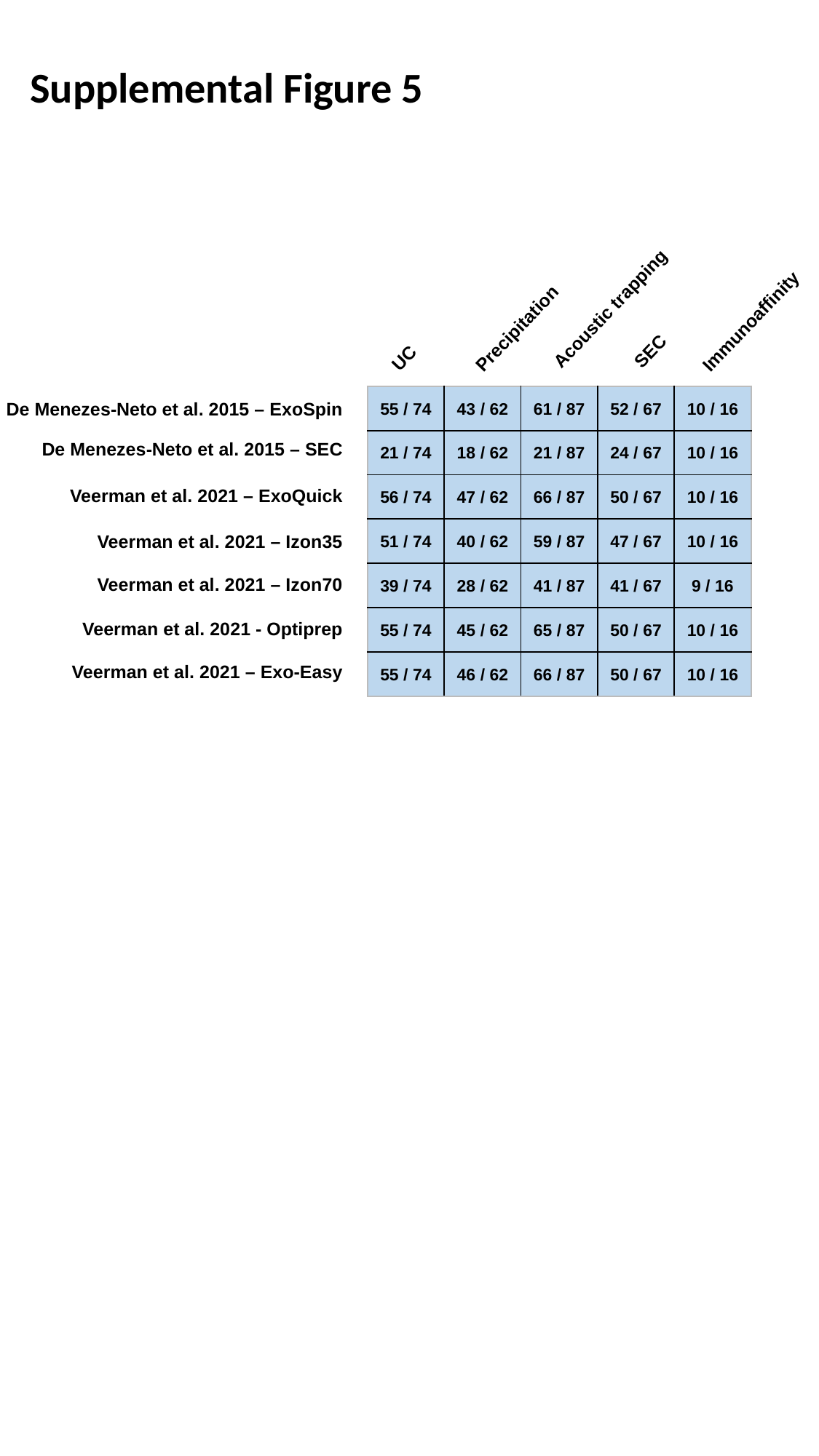

Supplemental Figure 5
Acoustic trapping
SEC
UC
Precipitation
Immunoaffinity
| 55 / 74 | 43 / 62 | 61 / 87 | 52 / 67 | 10 / 16 |
| --- | --- | --- | --- | --- |
| 21 / 74 | 18 / 62 | 21 / 87 | 24 / 67 | 10 / 16 |
| 56 / 74 | 47 / 62 | 66 / 87 | 50 / 67 | 10 / 16 |
| 51 / 74 | 40 / 62 | 59 / 87 | 47 / 67 | 10 / 16 |
| 39 / 74 | 28 / 62 | 41 / 87 | 41 / 67 | 9 / 16 |
| 55 / 74 | 45 / 62 | 65 / 87 | 50 / 67 | 10 / 16 |
| 55 / 74 | 46 / 62 | 66 / 87 | 50 / 67 | 10 / 16 |
De Menezes-Neto et al. 2015 – ExoSpin
De Menezes-Neto et al. 2015 – SEC
Veerman et al. 2021 – ExoQuick
Veerman et al. 2021 – Izon35
Veerman et al. 2021 – Izon70
Veerman et al. 2021 - Optiprep
Veerman et al. 2021 – Exo-Easy

## Slide 7
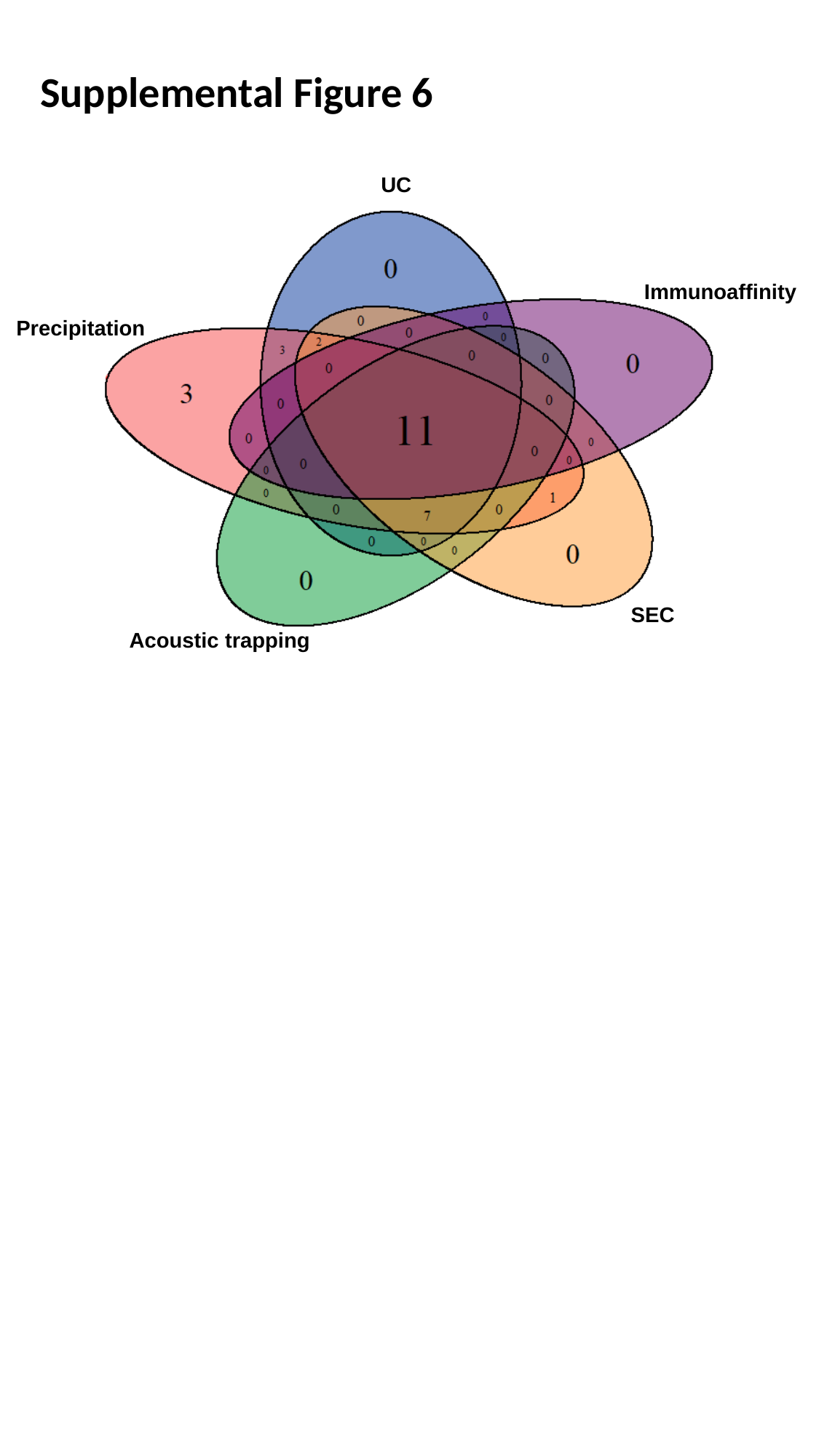

Supplemental Figure 6
UC
Immunoaffinity
Precipitation
SEC
Acoustic trapping

## Slide 8
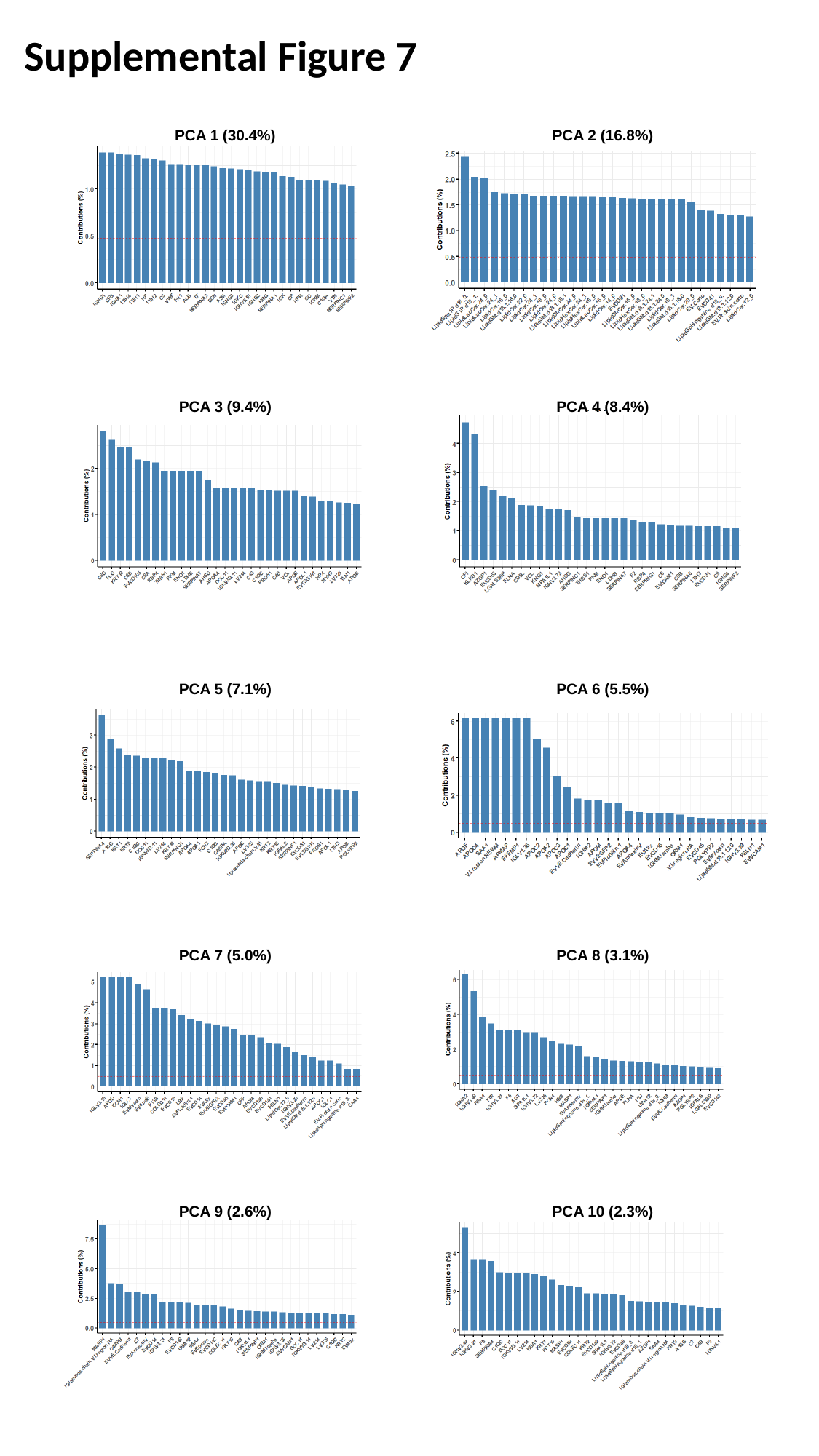

Supplemental Figure 7
PCA 1 (30.4%)
PCA 2 (16.8%)
PCA 3 (9.4%)
PCA 4 (8.4%)
PCA 5 (7.1%)
PCA 6 (5.5%)
PCA 7 (5.0%)
PCA 8 (3.1%)
PCA 9 (2.6%)
PCA 10 (2.3%)

## Slide 9
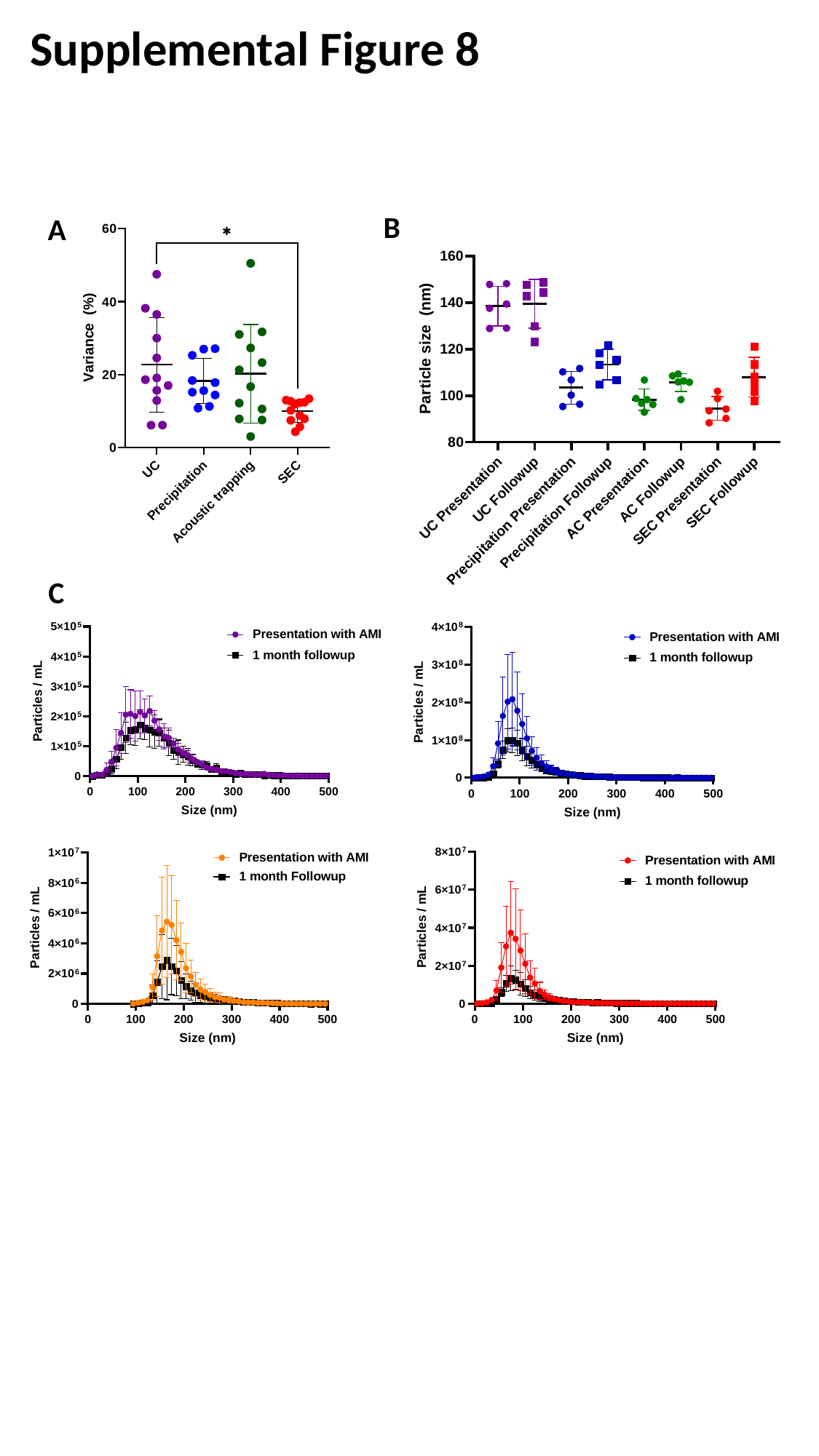

Supplemental Figure 8
B
A
C

## Slide 10
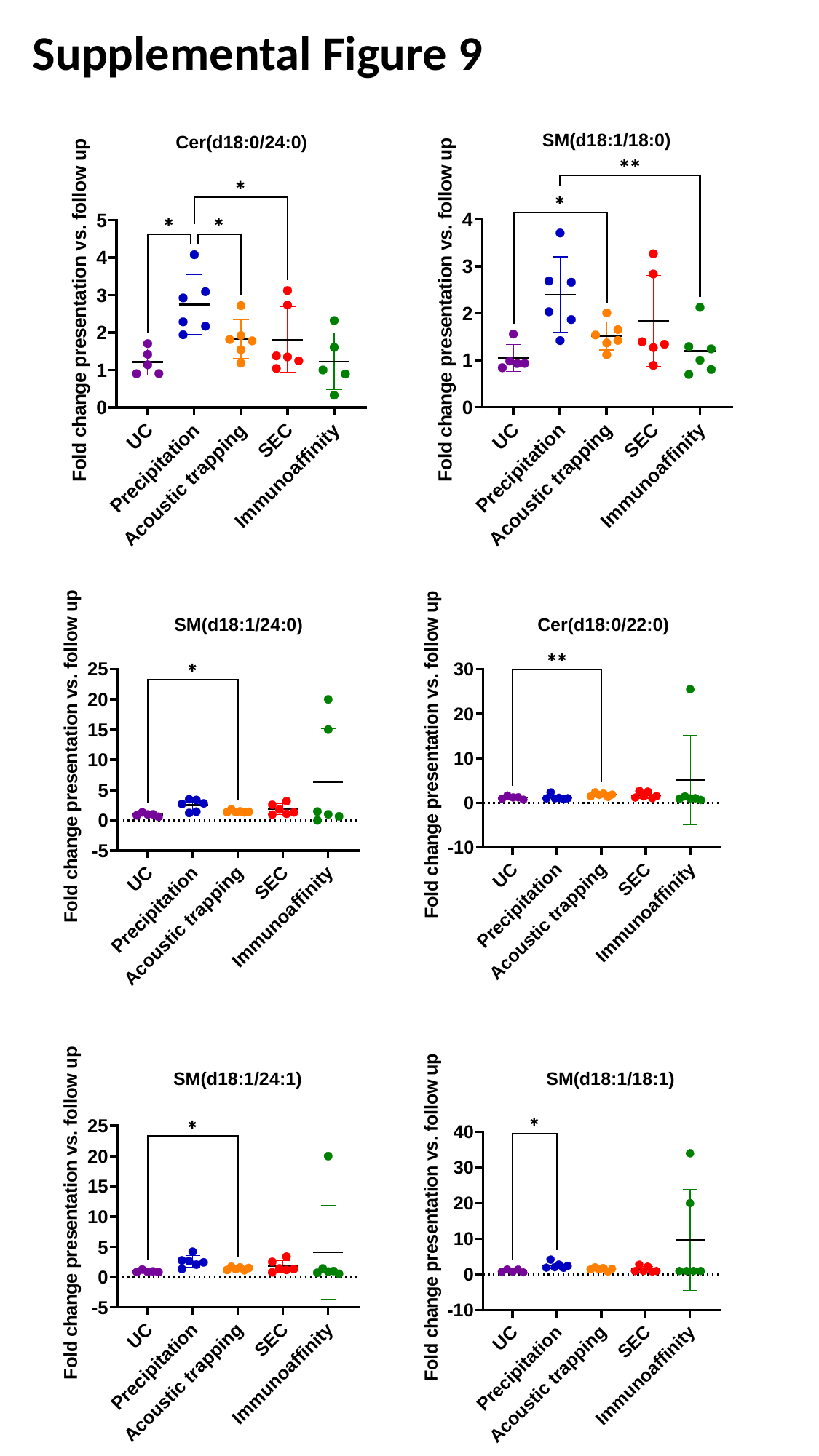

Supplemental Figure 9
SM(d18:1/18:0)
Cer(d18:0/24:0)
SM(d18:1/24:0)
Cer(d18:0/22:0)
SM(d18:1/24:1)
SM(d18:1/18:1)

## Slide 11
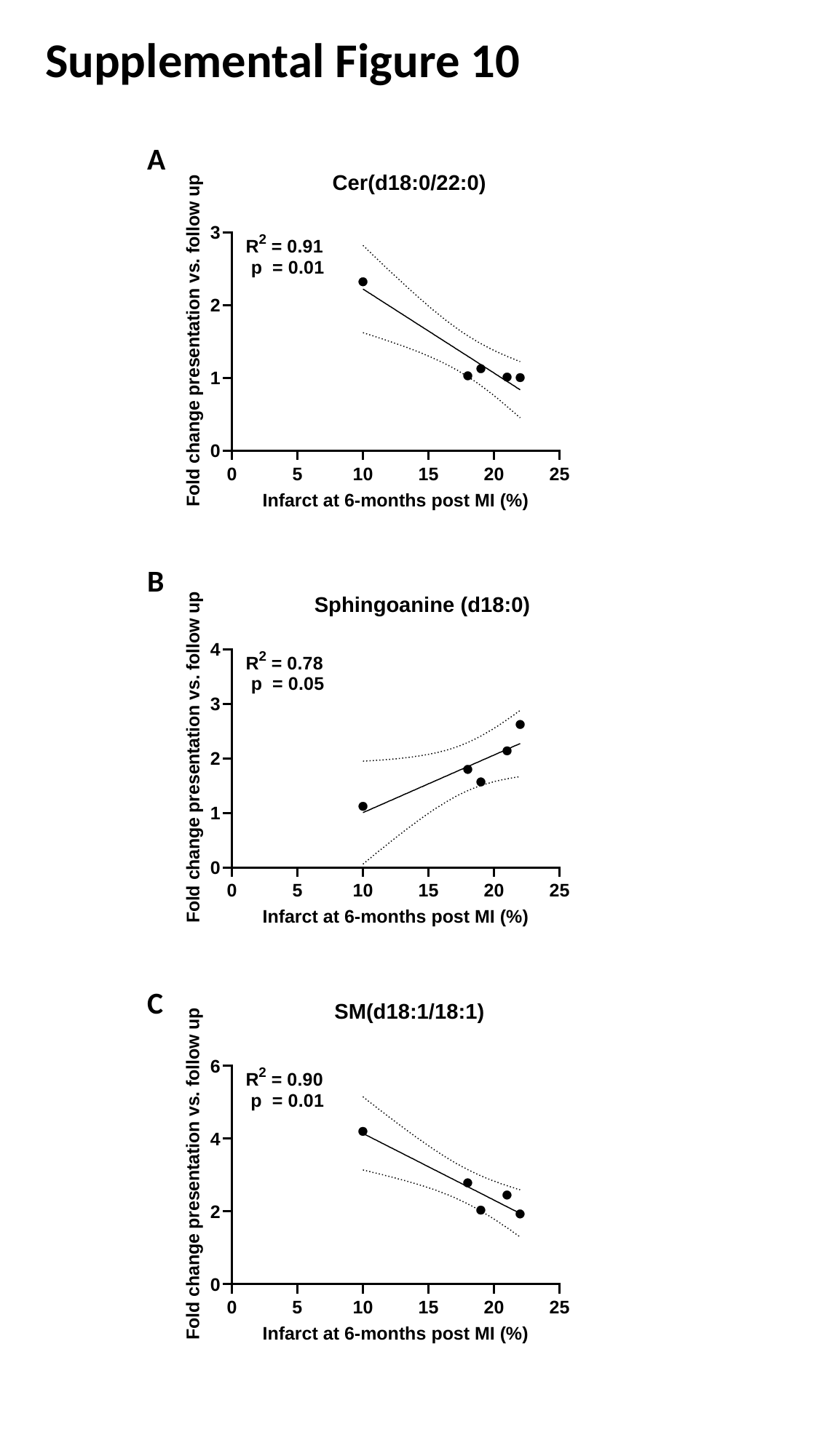

Supplemental Figure 10
A
Cer(d18:0/22:0)
B
Sphingoanine (d18:0)
C
SM(d18:1/18:1)
